# Critical Role of TLR2 and MyD88 for Functional Response of Macrophages to a Group IIA-Secreted Phospholipase A_2_ from Snake Venom

**DOI:** 10.1371/journal.pone.0093741

**Published:** 2014-04-09

**Authors:** Elbio Leiguez, Karina Cristina Giannotti, Vanessa Moreira, Márcio Hideki Matsubara, José María Gutiérrez, Bruno Lomonte, Juan Pablo Rodríguez, Jesús Balsinde, Catarina Teixeira

**Affiliations:** 1 Laboratório de Farmacologia, Instituto Butantan, São Paulo, Sao Paulo State, Brazil; 2 Instituto Clodomiro Picado, Facultad de Microbiología, Universidad de Costa Rica, San José, San José, Costa Rica; 3 Laboratorio de Investigación en Proteínas – FACENA-Universidad Nacional del Nordeste, Corrientes, Corrientes, Argentina; 4 The eicosanoid research division, Instituto de Biología y Genética Molecular, Valladolid, Valladolid, Spain; Fundação Oswaldo Cruz, Brazil

## Abstract

The snake venom MT-III is a group IIA secreted phospholipase A_2_ (sPLA_2_) enzyme with functional and structural similarities with mammalian pro-inflammatory sPLA_2_s of the same group. Previously, we demonstrated that MT-III directly activates the innate inflammatory response of macrophages, including release of inflammatory mediators and formation of lipid droplets (LDs). However, the mechanisms coordinating these processes remain unclear. In the present study, by using TLR2^−/−^ or MyD88^−/−^ or C57BL/6 (WT) male mice, we report that TLR2 and MyD88 signaling have a critical role in MT-III-induced inflammatory response in macrophages. MT-III caused a marked release of PGE_2_, PGD_2_, PGJ_2_, IL-1β and IL-10 and increased the number of LDs in WT macrophages. In MT-III-stimulated TLR2^−/−^ macrophages, formation of LDs and release of eicosanoids and cytokines were abrogated. In MyD88^−/−^ macrophages, MT-III-induced release of PGE_2_, IL-1β and IL-10 was abrogated, but release of PGD_2_ and PGJ_2_ was maintained. In addition, COX-2 protein expression seen in MT-III-stimulated WT macrophages was abolished in both TLR2^−/−^ and MyD88^−/−^ cells, while perilipin 2 expression was abolished only in MyD88^−/−^ cells. We further demonstrated a reduction of saturated, monounsaturated and polyunsaturated fatty acids and a release of the TLR2 agonists palmitic and oleic acid from MT-III-stimulated WT macrophages compared with WT control cells, thus suggesting these fatty acids as major messengers for MT-III-induced engagement of TLR2/MyD88 signaling. Collectively, our findings identify for the first time a TLR2 and MyD88-dependent mechanism that underlies group IIA sPLA_2_-induced inflammatory response in macrophages.

## Introduction

Secreted phospholipases A_2_ (sPLA_2_s) are enzymes that hydrolyse glycerophospholipids at the *sn-2* position of the glycerol backbone in a calcium-dependent manner, releasing fatty acids and lysophospholipids. These enzymes are members of the PLA_2_ superfamily, which is widely distributed in nature, and 15 groups and subgroups have been described, including five categories termed secreted PLA_2_, cytosolic PLA_2_, Ca^2+^ independent PLA_2_, the platelet-activating factor acetylhydrolase, and lysosomal PLA_2_ [1]. Among them, group IIA sPLA_2_ includes mammalian inflammatory-type and Viperid snake venom sPLA_2_s [2], [3]. Besides their relevant role in cell membrane physiology, mammalian group IIA sPLA_2_s are recognized as important paracrine and autocrine players in inflammatory processes by their ability to release fatty acids from cell membranes, leading to subsequent production of pro-inflammatory mediators such as prostaglandins, leukotrienes [1]. Accordingly, sPLA_2_ enzymes, particularly group IIA sPLA_2_s, have been implicated in the pathophysiology of several inflammatory diseases, such as atherosclerosis, inflammatory bowel disease, asthma and arthritis [4], [5], [6].

Besides structural homology, snake venom group IIA sPLA_2_s share functional features with mammalian group IIA sPLA_2_s, owing to their ability to induce inflammatory events in both *in vivo* and *in vitro* experimental models [7]. sPLA_2_s are abundant components in the proteomes of snake venoms from *Bothrops* genus [8] and have been associated with the prominent local inflammatory effects induced by these venoms. An Asp49 sPLA_2_, named myotoxin-III (MT- III), has been purified from *Bothrops asper* snake venom [9]. This group IIA sPLA_2_ has been reported to induce inflammatory events in *in vivo* experimental models [10], [11]. Furthermore, this venom sPLA_2_ exhibits the ability to directly activate inflammatory functions in isolated macrophages, such as increased phagocytic capacity, release of cytokines [11], [12], up-regulation of lipid mediator cascade, with release of prostaglandins [13], and increased formation of lipid droplets (LDs) [14], which are organelles implicated in the differentiation of macrophages into foam cells. However, the initial steps leading to this sPLA_2_-induced activation of macrophages are still unknown.

Macrophages are pivotal cells in innate immune responses. Activation of these cells initiates cascades of events that lead to acute inflammatory processes and the switch from the acute to the resolutive phase of inflammation, by releasing resolutive mediators, such as PGJ_2_ and IL-10 [15], [16]. In addition, macrophages are relevant cells in the development of many chronic inflammatory diseases, such as atherosclerosis. Macrophages loaded with LDs, termed foam cells, constitute an early hallmark of atherosclerotic lesion formation and a major cell source of inflammatory mediators [17], [18].

The macrophage innate response is initiated by activation of diverse membrane recognition receptors, such as the Toll-like receptors (TLRs). Nowadays, at least 13 TLRs have been described, each of them with a degree of specificity for various endogenous and exogenous ligands, eliciting different, but sometimes overlapping, immune responses [19], [20]. In inflammatory processes, recognition of pathogen-associated molecular patterns (PAMPs) by TLRs except TLR3 activates cell-signaling pathways mediated by the MyD88 [20]. Other kinds of stimuli, named endogenous damage-associated molecular patterns (DAMPs), are also capable of activating MyD88-dependent innate immune signaling by TLRs [21]. Some of the well- characterized DAMPs include: fibronectins [22], heat-shock proteins [23], pro-inflammatory cytokines from the monocyte-macrophage system [24], and free fatty acids (FFA) [25]. Among the TLRs, TLR2 constitutes a molecular link between elevated circulating FFA and inflammation [26]–[28]. TLR2 binds lipid-based structures via their long-chain saturated FFA moieties [25], [29], triggering inflammatory responses through MyD88 signaling [30]. Although TLRs are critical receptors directing specific inflammatory programs in macrophages, the involvement of TLR2 and MyD88 adaptor molecule signaling pathways in the activation of macrophages by group IIA sPLA_2_ remains unknown. The understanding of the mechanism(s) involved in sPLA_2_s-induced activation of in a pivotal immune innate cell, such as the macrophage, shall provide valuable information on new targets to confront the inflammatory diseases where group IIA sPLA_2_s enzymes are involved.

In the present study the involvement of TLR2 and MyD88 adaptor molecule in the signaling cascade (inflammatory signaling) elicited by MT-III in macrophages was investigated. To test this possibility, the effects of MT-III in macrophages from wild-type (WT) C57BL/6 mice, TLR2 gene knockout (TLR2^−/−^) mice and MyD88 gene knockout (MyD88^−/−^) were evaluated in terms of PGE_2_, PGD_2_, PGJ_2_, IL-1β and IL-10 biosynthesis, LDs formation, COX-2 and perilipin-2 expression and release of free fatty acids. Herein, we present the first evidence that TLR2 and MyD88 adaptor molecule are implicated in the inflammatory response triggered by a group IIA sPLA_2_ enzyme in macrophages. Engagement of TLR2/MyD88 signaling pathway by the venom sPLA_2_ MT-III coordinates events related to both initiation and resolution of the inflammatory response in macrophages. Our observations also suggest that saturated free fatty acids are major agonists for engagement of TLR2/MyD88 pathway induced by this group IIA sPLA_2_.

## Materials and Methods

### Ethics Statement

This study includes experiments with macrophages collected from pathogen-free 5-week-old wild type (WT), MyD88^−/−^ and TLR2^−/−^ mice (all on a C57BL/6 genetic background). These animals were bred in animal facilities at the University of São Paulo and were housed in a temperature-controlled room (22–24°C) with a 12 h light-dark cycle and fresh water and food ad libitum until used. All experimental procedures involving these animals were performed in accordance with the procedures laid down by the Universities Federation for Animal Welfare and were approved by the Animal Experimentation Ethics Committee of University of São Paulo (São Paulo, Brazil; Reference No 94/2010).

### Chemicals and Reagents

Hema-3 stain from Biochemical Sciences (Swedesboro, NJ, USA). MTT and L-glutamine were obtained from USB (Cleveland, OH, USA). E-toxate LAL kit and mouse mAb anti-β-actin was purchased from Sigma-Aldrich (St. Louis, MO, USA) and guinea pig polyclonal antibody anti- mouse perilipin-2 (PLIN2) from Research Diagnostics (Flanders, NJ, USA). PGE_2_ and PGD_2_ enzyme immunoassay kits and polyclonal antibodies against COX-2 were purchased from Cayman Company, Ann Arbor, MI, USA. 15-deoxy-Δ^12,14^– prostaglandin J_2_ (PGJ_2_) and IL-10 and IL-1β enzyme immunoassay kits were purchased from Enzo Life Sciences (Ann Arbor, MI, USA) and eBioscience, Inc. (San Diego, CA, USA), respectively. Secondary antibodies, anti- mouse and anti-guinea pig, conjugated to HRP and nitrocellulose membrane, were obtained from GE Healthcare (Buckinghamshire, UK). Gentamicin was purchased from Schering-Plough (Whitehouse Station, NJ, USA) and DMSO from Amresco (Solon, OH, USA). RPMI 1640, thiocarbohydrazide, and Osmium tetroxide (OsO_4_) were purchased from Sigma-Aldrich and RPMI from Life Technologies (São Paulo, SP, Brazil). Chloroform and methanol (HPLC grade) were purchased from Fisher Scientific (Hampton, NH), and [1–14C] palmitic acid and oleic acid from Perkin Elmer (Waltham, MA). Silicagel thin layer chromatography plates were purchased from Macherey-Nagel (Düren, Germany). Thioglycolate was obtained from Merck (Darmstadt, Germany) and all salts used were purchased from Sigma-Aldrich.

### Phospholipase A_2_ (PLA_2_)

Aspartate-49 sPLA_2_ named MT-III (Uniprot accession no.: P20474) from *B. asper* venom was purified by ion-exchange chromatography on CM-Sephadex C-25, using a KCl gradient from 0 to 0.75 M at pH 7.0 as described [2], followed by RP-HPLC on a semipreparative C8 column (Vydac; 10×250 mm, 5 μm particle size) eluted at a flow rate of 2.5 ml/min with a gradient of acetonitrile (0–70%, containing 0.1% trifluoroacetic acid) over 30 min. Homogeneity was verified by SDS-PAGE, run under reducing conditions, in which a single band of ∼14 kDa was observed. The complete amino acid sequence of this enzyme has been described previously [31], [32]. The absence of endotoxin contamination in the MT-III batches used was demonstrated by performing the quantitative LAL test [33], which revealed undetectable levels of endotoxin (<0.125 EU/mL).

### Macrophages Harvesting and Stimulation

Animals were killed under CO_2_ atmosphere and cells were then harvested by washing peritoneal cavities with 3 mL apyrogenic phosphate buffered saline (PBS), pH 7.2. Aliquots of the washes were used for total cell counts in a Neubauer chamber, after dilution (1∶20, v/v) in Turk solution (0.2% crystal violet dye in 30% acetic acid). Differential cell counts were performed on smears stained with Hema3. More than 95% of the cell population consisted of macrophages, as determined by conventional morphological criteria. The remaining wash volumes were centrifuged at 129×*g* for 6 min (4°C), supernatants were discarded and cell pellets were appropriately diluted and maintained in serum-free RPMI 1640 medium, supplemented with 2 mM L-glutamine and 40 μg/mL gentamicin sulfate at 37°C with 5% CO_2_, and processed according to the experimental protocols used, as follows: 1) 1×10^6^ macrophages/well were seeded in 12 wells culture plates and maintained in culture medium for 24 h before stimulation, and then used for Western blotting analysis of COX-2 and perilipin-2 protein expression; 2) 2×10^5^ macrophages, harvested 4 days after i.p. injection of 1 mL 3% thioglycolate, were seeded on glass coverslips and incubated in culture medium for 30 min for adhesion, followed by washing with PBS, and then used for LDs evaluation; 3) 1×10^7^ macrophages were seeded in cell culture dish, maintained in culture medium for 18 h, and used for gas chromatography/mass spectrometry analysis of fatty acid methyl esters; 4) 2×10^5^ macrophages/well were seeded in 12 well culture plates, maintained with culture medium for 30 min for adhesion and then incubated with exogenous [1–14C] Oleic or [1–14C] Palmitic acid (1 μM; 0.25 μCi/ml) for 18 h at 37°C. Cells were then scraped with 0.1% Triton X-100 in PBS and supernatants were removed and total ^14^C-radioactivity levels were determined by scintillation counting. After each of the above described procedures, cells were stimulated with MT-III diluted in RPMI 1640 medium in a final concentration of 0.4 μM or RPMI 1640 medium alone (control) for selected periods of time and maintained at 37°C in a humidified atmosphere (5% CO_2_).

### Quantification of Eicosanoids and Cytokines

Prostaglandins (PGE_2_, PGD_2_ and PGJ_2_) and cytokines (IL-1β and IL-10) were quantified in supernatants from macrophages cultures by using commercially available enzyme immune assay kits, performed according to instructions of the manufacturer.

### Lipid Droplet (LD) Staining and Quantification

Analysis of LD numbers was performed in osmium-stained cells. In brief, macrophages adhered to glass coverslips were fixed in 4% paraformaldehyde in 0.1 M phosphate buffer (pH 7.2) for 15 min and stained with OsO_4_. The coverslips were then rinsed in 0.1 M phosphate buffer, stained with 1% OsO_4_ (30 min), rinsed in deionized H_2_O, immersed in 1.0% thiocarbohydrazide (5 min), rinsed again in 0.1 M phosphate buffer, re-stained with 1% OsO_4_ (3 min), rinsed with H_2_O, and then dried and mounted [42]. The morphology of the fixed cells was observed, and round intracellular osmiophilic structures were identified as LDs, which were then counted under phase-contrast microscopy using the 100×objective lens in 50 consecutively scanned leukocytes in each coverslip.

### Western Blotting for COX-2 and Perilipin-2

COX-2 and perilipin-2 protein levels in cultured macrophages were detected by Western blotting. Aliquots of MT-III-stimulated and non-stimulated cells (1.5×10^6^ cells) were lysed with 100 μL sample buffer (0.5 M Tris-HCl, pH 6.8, 20% SDS, 1% glycerol, 1 M β-mercapto ethanol, 0.1% bromophenol blue) and boiled for 10 min. Samples were resolved by SDS-PAGE on 10% bis-acrylamide gels overlaid with a 5% stacking gel. Proteins were then transferred to nitrocellulose membranes using a Mini Trans-Blot (Bio-Rad Laboratories, Richmond, CA, USA). The membranes were blocked for 1 h with 5% nonfat dry milk in Tris-buffered saline (TBS) (20 mM Tris, 100 mM NaCl, and 0.5% Tween 20, pH 7.2), and incubated with primary antibodies against COX-2 (1∶1000 dilution), perilipin-2 (1∶2000) and β-actin (1∶3000) for 1 h. They were then washed and incubated with the appropriate secondary antibody conjugated to horseradish peroxidase. The immunonereactive bands were detected by the entry-level peroxidase substrate for enhanced chemiluminescence (ECL), according to the instructions of the manufacturer (GE Healthcare). Band densities were quantified with a GS 700 densitometer (Bio- Rad Laboratories) using the image analysis software Molecular Analyst (Bio-Rad Laboratories).

### Gas Chromatography/mass Spectrometry Analysis of Fatty Acid Methyl Esters

A cell extract was used and, before extraction, 10 nmol of 1,2,3-triheptadecanoylglycerol was added as internal standard. Then, total lipids were extracted according to the Bligh and Dyer method [34], and glycerolipids were transmethylated with 500 μl of 0.5 M KOH in methanol for 60 min at 37°C. For neutralization, 500 μl of 0.5 M HCl were added and the extraction of fatty acid methyl esters was performed with 1 ml of n-hexane twice. Analysis of fatty acid methyl esters was carried out in a Agilent 7890A gas chromatograph coupled to an Agilent 5975C mass-­-selective detector operated in electron impact mode (EI, 70 eV), equipped with an Agilent 7693 autosampler and an Agilent DB23 column (60 m length×250 μm internal diameter×0.15 μm film thickness) under the following conditions: oven temperature was held at 50°C for 1 min, then increased to 175°C at a rate of 25°C/min, then increased to 215°C at a rate of 1.5°C/min, and the final ramp being reached at 235°C at a rate of 10°C/min. The final temperature was maintained for 5 min, and the run time was 39.67 min. Data analysis was carried out with the Agilent G1701EA MSD Productivity Chemstation software, revision E.02.00, and the quantification of fatty acid methyl esters was achieved by integration of chromatographic peaks comparing with authentic analytical standards [35], [36]. The fatty acids evaluated are indicated below in table 1.

**Table 1:** Fatty acid nomenclature.

Abbreviation	Systematic Name	Commom name
16_0	Hexadecanoic Acid	Palmitic acid
18_0	Octadecanoic Acid	stearic acid
18_1 n-9	9-Octadecenoic Acid	oleic acid
18_1 n-7	11-Octadecemoic Acid	cis-vaccenic acid
18_2 n-6	9,12-Octadecadienoic Acid	Linoleic acid
20_1 n-9	11-Eicosenoic Acid	Gondoic Acid
20_2 n-6	11,14-Eicosadienoic Acid	none
20_3 n-6	8,11,14-Eicosatrienoic Acid	Dihomo-gamma-linolenic acid
20_4 n-6	5,8,11,14-Eicosatetraenoic Acid	Arachidonic acid
22_5 n-6	4,7,10,13,16-Docosapentaenoic Acid	Osbond Acid
22_5 n-3	7,10,13,16,19-Docosapentaenoic Acid	Clupanodonic Acid
22_6 n-3	4,7,10.13.16,19-Docosahexaenoic Acid	Cervonic Acid

### Quantification of Fatty Acids (FA) Released

The cells were placed in serum-free medium and exposed to exogenous [^14^C] Oleic or [^14^C] Palmitic acid (1 μM; 0.25 μCi/ml) for 18 h at 37°C. Afterward, the cells were washed four times with PBS containing 0.5% albumin to remove the fatty acids that had not been incorporated, and next stimulated with MT-III or RPMI medium only (control) for 6 h. Cells were scraped with 0.1% Triton X-100 in PBS, supernatants were removed, and total ^14^C-radioactivity levels were determined by scintillation counting.

### Statistical Analysis

Data are expressed as the mean ± standard error of mean (S.E.M) of at least three animals. Multiple comparisons among groups were performed using one-way ANOVA and Tukey test as a post-test. Differences between experimental groups were considered significant for *P*-values <0.05. All statistic tests were performed using Prism version 5 software (GraphPad, San Diego, CA).

## Results

### Prostaglandin Production Induced by MT-III Requires Distinct TLR Signaling Pathways

**Figure 1:**
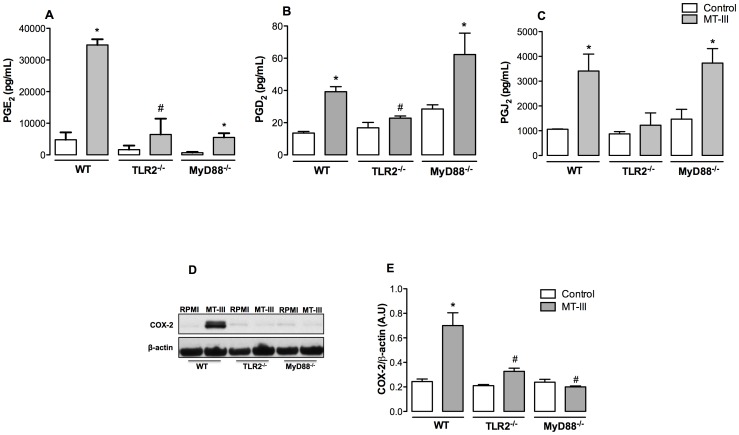
TLR2 and Myd88 signaling pathways are involved in MT-III-induced prostanoid biosynthesis and COX-2 protein expression. Wild type (WT) or TLR2^−/−^ or MyD88^−/−^ peritoneal macrophages were incubated with MT-III (0.4 μM) or RPMI (control) for 6 h. PGE_2_ (A), PGD_2_ (B) and PGJ_2_ (C) concentrations were quantified in culture supernatants by EIA commercial kits; (D) Western blotting immunoreactive bands of COX-2 and β-actin (loading control) representative of at least three samples/experimental group; (E) Densitometric analysis of immunoreactive COX-2 band intensities. Densities (in arbitrary units) were normalized with those of β-actin. Results are expressed as mean ± SEM from 3–6 animals. *p<0.05 as compared with control cells; ^#^ *p*<0.05 as compared with MT-III-stimulated cells.

Prostaglandins are produced in abundance at sites of inflammation and regulate many aspects of the inflammatory response. Prostaglandins such as PGE_2_ and PGD_2_ have well-defined roles in acute inflammation, such as the regulation of blood flow and edema formation, cytokine generation, and leukocyte migration [37], [38]. In contrast to these lipid mediators, PGJ_2_, which is a PGD_2_-derived prostanoid, has been described as a resolutive mediator, which is involved in the switch from the acute into the regenerative phases of inflammatory processes [16]. We have previously reported that the sPLA_2_ MT-III elicits the biosynthesis of prostaglandins in isolated peritoneal macrophages [13]. These relevant cells of the innate immune system sense danger signals via recognition receptors such as TLRs, and then initiate a coordinated inflammatory response. However, whether TLR pathways, particularly TLR2 and MyD88 adaptor molecule activation, are required for this and other MT-III-induced effects in macrophages is unknown, and deserve further investigation. Therefore, we initially evaluated the involvement of TLR2 and MyD88 signaling in MT-III-induced PGE_2_, PGD_2_ and PGJ_2_ production in TLR2- and MyD88- deficient macrophages. Results presented in Figure 1 (A–C) show that PGE_2_ levels were significantly increased in MT-III-stimulated macrophages collected from WT mice. This MT-III- induced PGE_2_ release was impaired in macrophages obtained either from TLR2^−/−^ or MyD88^−/−^mice. Notably, sPLA_2_ MT-III failed to induce PGD_2_ and PGJ_2_ production in TLR2^−/−^, but did induce production of these mediators in MyD88^−/−^ macrophages when compared with MT-III- stimulated WT cells. These observations prompted us to consider that TLR2 signaling is engaged for the biosynthesis of PGE_2_, PGD_2_ and PGJ_2_ in macrophages under stimuli by MT-III. However, the TLR2 via MyD88 adaptor molecule pathway operates only for biosynthesis of PGE_2_, whereas production of PGD_2_ and PGJ_2_ occurs through an adaptor molecule other than MyD88. Of note, these findings further suggest that upon activation by the sPLA_2_ MT-III, TLR2 and MyD88 signaling pathways can coordinate macrophage responses related to both the development and resolution steps of inflammation.

### Up-regulation of COX-2 Protein Expression Induced by MT-III is Dependent on TLR2 via MyD88 Signaling Pathway

Activation of TLR2 via MyD88-mediated signaling leads to activation of transcription factors such as NF-*k*B, AP-1 and IRF, which in turn play an important role in gene transcription of several molecules involved in the inflammatory response, such as cytokines and cyclooxygenase-2 (COX-2) [39]–[42]. Prostaglandins are produced by cyclooxygenases, which occur in constitutive (COX-1) and inducible (COX-2) forms [6]. We have previously demonstrated that MT-III up-regulates COX-2 protein expression in peritoneal macrophages and that this isoform is responsible for the increased levels of prostaglandins induced by this sPLA_2_ [13]. However, the involvement of TLR2 and MyD88 signaling pathway in this process is unknown. The potential role of TLR2 and MyD88 signaling in MT-III-induced COX-2 protein expression was then analyzed. Figure 1 (D) shows that TLR2^−/−^ and MyD88^−/−^ macrophages stimulated by MT-III did not present the increased COX-2 protein expression seen in MT-III-stimulated WT macrophages. These data clearly indicate that TLR2 and MyD88 are essential to this effect induced by the sPLA_2_ MT-III, constituting a key step in the onset of MT-III-induced activation of macrophages. Participation of MyD88 signaling in this MT-III-induced effect is consistentwith previous reports by others that activation of this adaptor molecule signals for gene expression of COX-2 [19], [43].

### TLR2 via MyD88 Signaling Pathway is Required for MT-III-induced Production of IL-1β and IL-10 in Macrophages

**Figure 2:**
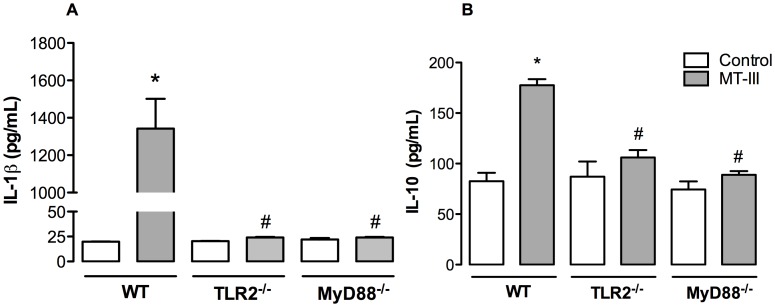
TLR2 and MyD88 signaling pathways are relevant for MT-III-induced IL-1β and IL-10 release. Wild type (WT) or TLR2^−/−^ or MyD88^−/−^ peritoneal macrophages were incubated with MT-III (0.4 μM) or RPMI (control) for 6 h. IL-1β (A) and IL-10 (B) concentrations were quantified in culture supernatants by EIA commercial kits. Results are expressed as mean ± SEM from 3–6 animals. *p<0.05 as compared with control group; ^#^ *p*<0.05 as compared with MT-III- stimulated WT cells.

IL-1β and IL-10 are relevant mediators involved in triggering and down-regulation of inflammatory processes, respectively [44], [45]. Despite the distinct roles played by these two cytokines in inflammation, the production of these mediators has been shown to be regulated by TLR2 and MyD88 signaling [46]. The ability of MT-III to induce the release of inflammatory cytokines, such as IL-1β, has been previously demonstrated [12]. However, whether this MT-III- induced event is coordinated by TLR2 and MyD88 pathways is unknown. Moreover, neither the ability of MT-III to induce release of IL-10 by macrophages nor the participation of TLR2 and MyD88 pathways in this effect has been investigated. We therefore explored the potential role of TLR2 and MyD88 signaling in MT-III-induced synthesis of these cytokines. As demonstrated in Figure 2 (A–B), stimulation of WT macrophages with MT-III induced a significant release of IL- 1β and IL-10 from these cells. However, stimulation of TLR2^−/−^ and MyD88^−/−^ macrophages by MT-III did not result in an increased release of these two cytokines as compared with MT-III- stimulated WT macrophages. These data confirm previous observations on the ability of MT-III to induce the release of IL-1β and additionally evidenced the capacity of this sPLA_2_ to induce the release of IL-10. Furthermore, these data implicate the TLR2 via MyD88 signaling pathway in the generation of IL-1β and IL-10 induced by MT-III in macrophages.

### TLR2 via MyD88 is a Critical Pathway for MT-III-induced LD Formation

**Figure 3:**
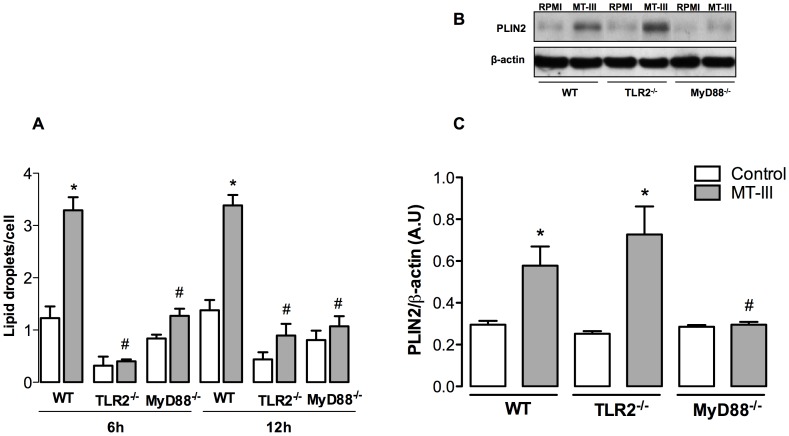
TLR2 and MyD88 are required for lipid droplet formation, but only MyD88 is relevant to perilipin-2 protein expression induced by MT-III. Wild type (WT) or TLR2^−/−^ or MyD88^−/−^ peritoneal macrophages were incubated either with MT-III (0.4 μM) or RPMI (control) for 6 or 12 h. (A) Lipid droplets numbers; (B) Western blotting immunoreactive bands of perilipin-2 (PLIN2) and β-actin (loading control); (C) Densitometric analysis of immunoreactive PLIN2 band intensities. Densities (in arbitrary units) were normalized with those of β-actin. Results are expressed as mean ± SEM from 3–6 animals. *p<0.05 as compared with control group; #*p*<0.05 as compared with MT- III- stimulated WT cells.

It has been demonstrated that LDs formation in leukocytes is a highly regulated event that depends on the interaction of cellular receptors with their ligands. These organelles were shown to be involved in the synthesis of inflammatory mediators and are markers of leukocyte activation [47], [48]. Moreover, the role of TLRs-mediated pathogen recognition and activation in the mechanism of LDs formation has been demonstrated by others [48], [49]. We have recently shown that MT-III induces the formation of LDs in macrophages [14]. However, the mechanisms underlying this MT-III-induced effect remain unclear. To further understand this issue we investigated the involvement of TLR2 and MyD88 signaling. As demonstrated in Figure 3A, the sPLA_2_ MT-III failed to induce LDs formation in macrophages from both TLR2^−/−^ and MyD88^−/−^ mice when compared with macrophages from WT mice, at both time intervals evaluated. These findings identify TLR2 via MyD88 adaptor molecule as an essential signaling pathway regulating LD biogenesis induced by the venom sPLA_2_ MT-III in macrophages. Considering that LD-laden macrophages constitute the foam cells seen at the sites of atherosclerotic lesion, in which levels of the sPLA_2_s are increased, our present findings suggest that activation of TLR2/MyD88-mediated signaling pathway is likely to be an important mechanism that accounts for foam cell formation stimulated by group IIA sPLA_2_s.

### Requirement of TLR2 and/or MyD88 Signaling for MT-III-induced Perilipin-2 up- Regulation and Mobilization

**Figure 4:**
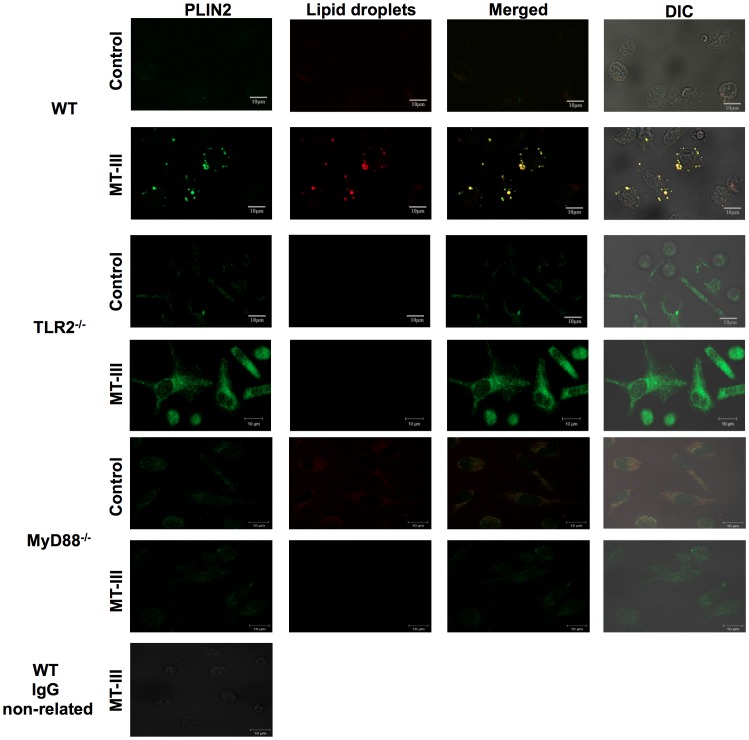
TLR2 and MyD88 are essential for MT-III-induced perilipin-2 (PLIN2) subcellular distribution. Wild Type (WT), TLR2^−/−^ or MyD88^−/−^ peritoneal macrophages were incubated with RPMI (control) or MT-III (0.4 μM) for 6 h and labeled for both lipid droplets (fluorescent Nile red) and anti-PLIN2 (FITC-conjugated immunocomplex). Merged image shows localization of PLIN2 to lipid droplets in WT macrophages. Cell nuclei were stained with propidium iodide. IgG control was included and showed negative stain. The micrographs are representative of at least three samples/experimental group.

Perilipin-2 has long been reported as a pivotal protein for neutral lipid accumulation that serves as scaffolding during LD formation in diverse cell types, including macrophages [50], [51]. This protein is constitutively and ubiquitously expressed in macrophages as a major component of intracellular LDs [50], [52]–[54]. Moreover, the expression of perilin-2 can be up-regulated during inflammatory processes [51], [55], [56]. Consistent with these properties, perilipin-2 has been used as a marker of lipid loading and LD assembly in inflammatory cells [57]. Accumulating evidence indicates that activation of the Toll like receptors triggers foam cell formation and increase of perilipin-2 protein expression [49], [58], [59]. In macrophages, MT-III has been shown to up- regulate perilipin-2 protein expression and to trigger mobilization of constitutive perilipin-2 to form new LDs in macrophages [14]. On this basis, we assessed the involvement of TLR2 and MyD88 in perilipin-2 protein expression induced by MT-III in macrophages for 6 h. As demonstrated in Figure 3B, following MT-III stimulation of WT macrophages, there is a significant increase of perilipin-2 protein expression in comparison with RPMI-stimulated WT macrophages. This increased protein expression was observed in TLR2^−/−^ macrophages stimulated by MT-III when compared with WT macrophages. Conversely, MyD88^−/−^ macrophages stimulated by this sPLA_2_ were unable to up-regulate perilipin-2 protein expression as compared to WT macrophages. These data indicate that perilipin-2 protein expression induced by MT-III is dependent on MyD88 pathway, but not on TLR2 activation. Next we evaluated the potential role of TLR2 and MyD88 signaling in subcellular mobilization of perilipin-2 induced by MT-III. As illustrated in Figure 4, WT macrophages stimulated by MT-III exhibited strong fluorescent staining (green) for perilipin-2, with a punctate cytoplasmic pattern, which was absent in RPMI-stimulated control cells. Fluorescent Nile Red-labeled LDs were also visualized after MT-III-induced stimulation and were virtually absent in non-stimulated WT macrophages. After stimulation with MT-III, cytoplasmic-stained perilipin-2 matched perfectly with Nile Red- marked neutral lipid inclusions. As expected, no significant staining was detected in WT control macrophages. However, this phenomenon was not observed in macrophages from TLR2^−/−^ or MyD88^−/−^ mice, demonstrating that perilipin-2 subcellular distribution induced by MT-III is dependent on TLR2 and MyD88 signaling pathway in a tight correlation with the regulation of MT-III-induced LD formation by TLR2/MyD88 pathway.

### MT-III Releases Free Fatty Acids from Macrophages

**Figure 5:**
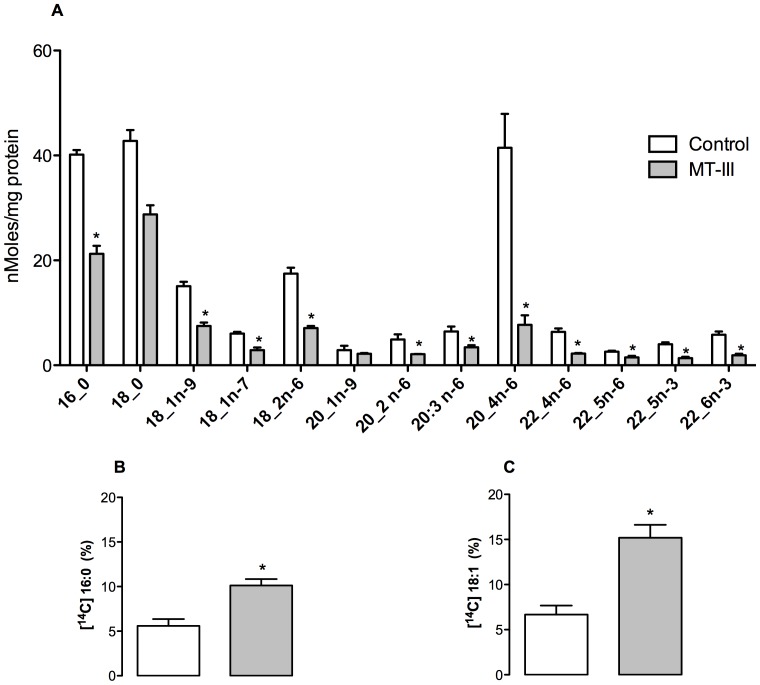
MT-III affects macrophage fatty acid content causing the release of palmitic and oleic acids. (A) The profile of major fatty acids in peritoneal macrophages from WT mice stimulated with MT-III (0.4 μM) (gray bars) or RPMI (control) (open bars) for 6 h was determined by GC- MS after converting the glyceryl fatty acid into fatty acid methyl esters. (B) Peritoneal macrophages were incubated with 1 μM [^14^C]palmitic acid or (C) [^14^C] oleic acid for 18 h. Afterward, [^14^C]palmitic acid or [^14^C] oleic acid incorporation was measured in macrophages and their supernatants 6 h after stimulation with either MT-III or RPMI (control) as described in Materials and Methods. The ^14^C radioactivity incorporated is expressed as a percentage of the radioactivity originally added to the cells. Results are expressed as means ± SEM from 3–6 animals. *p<0.05 as compared with the corresponding control.

To better understand the mechanisms underlying activation of TLR2 and MyD88 signaling by MT-III in macrophages, we analyzed the ability of this sPLA_2_ to generate free fatty acids, which are recognized as efficient agonists of TLRs, mainly TLR2 [25], [28], [29], [60]. The total fatty acid profile was then analyzed in MT-III-stimulated macrophages by measuring fatty acid methyl esters using gas chromatography mass spectrometry coupled. Figure 5A shows that stimulation of WT macrophages with the sPLA_2_ MT-III resulted in a remarkable reduction of fatty acids presumably due to the action of this enzyme on membrane phospholipids. Large decreases were observed in palmitic, stearic, oleic, and particularly in arachidonic acid, which clearly generated a pool of intracellular free fatty acids, that must be reorganized in order to keep intact the cellular homeostasis. To accomplish this, two of the possible underlying mechanisms are: 1) lipid droplet formation and 2) excretion of fatty acids into the extracellular environment. In order to test this latter hypothesis, we previously added labeled free fatty acids (palmitic and oleic acid) to the peritoneal macrophage culture and let them to incorporate; then we determined the blink per minute in a radioactivity counter. Figure 5B shows that higher amounts of palmitic and oleic acids were found in culture supernatants of MT-III-stimulated WT macrophages than in controls, suggesting that these FFA may consequently stimulate the Toll like receptors by an autocrine mechanism.

## Discussion

Group IIA secreted PLA_2_ enzymes are considered important players in inflammatory process owing to their ability to induce the biosynthesis of second messengers that stimulate innate responses of inflammatory cells, including macrophages. As TLRs are recognized to trigger a pro-inflammatory program in macrophages and respond to exogenous molecules and host-derived endogenous ligands [20], [61], in the present study we investigated the participation of TLR2 and MyD88 adaptor molecule in the stimulatory effects of the venom sPLA_2_ MT-III in macrophages.

The data presented herein indicate a major role for TLR2 in MT-III-induced production of inflammatory mediators by macrophages, since the production of PGE_2_, PGD_2_ and PGJ_2_ induced by MT-III was abrogated in TLR2^−/−^ macrophages, indicating the engagement of TLR2 upon stimulus by this sPLA_2_. To our knowledge, this is the first demonstration that TLR2 pathway is activated by a secreted PLA_2_ enzyme leading to biosynthesis of lipid mediators. Reports from literature have shown that endogenous Group V and Group X sPLA_2_s are capable to amplify signaling through TLR2 and TLR4/MyD88 in cells stimulated by the pathogen- activated molecular patterns Pam3CSK4 and LPS, leading to biosynthesis of eicosanoids in mast cells and macrophages, respectively [31], [69]. Furthermore, we found that the adaptor molecule MyD88 plays a role in MT-III-induced production of PGE_2_, since the release of this mediator was abrogated in MyD88^−/−^ macrophages stimulated by MT-III. However, the increment of PGD_2_ and PGJ_2_ induced by MT-III was not altered in MyD88^−/−^ macrophages, suggesting that their synthesis is triggered through an adaptor molecule other than MyD88, in the present experimental condition. This is the first demonstration that a secreted group IIA PLA_2_ directly activates macrophages to release PGJ_2_. Noteworthy, the present findings also evidence, for the first time, that the production of a resolutive mediator is regulated by the TLR2 pathway.

A previous report of our group demonstrated that, similarly to mammalian GIIA sPLA_2_s [70], MT-III up-regulates COX-2 protein expression, a critical enzyme for PGs production in macrophages [13]. We herein extended the understanding of this mechanism by showing that MT-III up-regulates COX-2 expression via TLR2 and MyD88 signaling pathways in macrophages, based on the inability of TLR2^−/−^ and MyD88^−/−^ macrophages to express COX-2 in response to MT-III stimulus. Taking these data into account, and considering results showing that MyD88 adaptor molecule is involved in MT-III-induced production of PGE_2_, but not PGD_2_ or PGJ_2_, it is suggested that COX-2 is the major PGE_2_ biosynthetic pathway, whereas production of PGD_2_ and PGJ_2_ may be associated to both COX-2 and COX-1 biosynthetic pathways. Consistent with this hypothesis, a previous report has shown the ability of MT-III to induce an increment in the activity of COX-1 along with expression of COX-2 in macrophages [13].

The ability of sPLA_2_s, including MT-III, to induce the release of cytokines has been previously demonstrated [12], [71]. The data presented herein demonstrate that this sPLA_2_ induces the release of IL-10 and IL-1β in a process mediated by TLR2/MyD88 signaling pathway. These findings also bring additional evidence that engagement of TLR2/MyD88 signaling pathway coordinates both the acute and resolutive responses in macrophages upon stimulation by the sPLA_2_ MT-III. Furthermore, increased IL-10 biosynthesis, observed in MT-III-stimulated WT macrophages, suggests that in our experimental conditions this cytokine regulates the synthesis of PGE_2_ via TLR2/MyD88 pathway activation, as a tight regulation of PGE_2_ biosynthesis by IL-10 has been previously demonstrated in macrophages [62]–[64].

LDs are cytoplasmic inclusions found in numerous immune cells associated with inflammation [51], [54], [65], such as foam macrophages, which are major cells of atherosclerotic lesions [66]. We recently demonstrated that MT-III induces LDs formation, recruitment and increased expression of the LD scaffold protein perilipin-2 in macrophages [14]. The results obtained herein demonstrate, for the first time, that TLR2 and MyD88 are essential elements for LD formation induced by MT-III, since TLR2^−/−^ and MyD88^−/−^ macrophages failed to respond with increased numbers of LDs. These results are in agreement with previous reports by others showing that TLR2/MyD88 pathway is involved in LDs formation in leukocytes [47]–[49]. On the other hand, our data further demonstrate that TLR2 deletion does not affect the ability of MT-III to induce a significant increase of perilipin-2 protein expression, but inhibits its capacity to mobilize constitutive perilipin-2 in the cytosol, evidencing that although not signaling for up regulation of perilipin-2 expression, this receptor is engaged for its recruitment, triggering an important mechanism for LD formation. In contrast, MyD88 deleted cells blocked MT-III- induced expression and mobilization of perilipin-2, implying the participation of this adaptor molecule in this effect. Therefore, activation of a TLR other than TLR2 is involved in MT-III- induced expression of perilipin-2. To our knowledge, this is the first demonstration of participation of TLR2 and MyD88 pathways in the mechanisms leading to LDs formation induced by group IIA sPLA_2_, and that MyD88 is a crucial adaptor molecule mediating perilipin-2 expression and mobilization induced by this class of enzymes. Experimental studies have previously demonstrated that TLRs, particularly TLR2 via MyD88-dependent pathway, have relevant roles in the differentiation of recruited monocytes into lipid-laden macrophages (foam cells), and ultimately to the development of atherosclerotic lesions [67], [68]. Considering the reported increased expression of group IIA sPLA_2_s at the site of atherosclerotic lesions [9], our present findings may shed light on the early mechanisms involved in the ability of sPLA_2_s to induce formation of foam macrophages.

The mechanisms by which MT-III activates TLR2 were not presently investigated. It has been shown that a free fatty acid (FFA)-rich microenvironment leads to the activation of TLR2 [28], [60]. Moreover, there are data demonstrating that products of cleavage of membrane phospholipids by sPLA_2_s, including saturated fatty acids, lysophosphatidylcholine and lysophosphatidylserine, are able to activate this receptor [60], [72], [73], [74]. In this context, we herein demonstrate that MT-III reduced the levels of saturated FFA, such as palmitic and stearic acid, as well as monounsaturated and polyunsaturated fatty acids in macrophages. Decreased levels of both palmitic and oleic acids correlated with increased amounts of these fatty acids in the extracellular medium, indicating that they are released from macrophages upon the action of MT-III, thus allowing their interaction with surface membrane receptors. On this basis, we suggest that, in the present experimental conditions, saturated FFAs resulting from the catalytic action of MT-III on membrane phospholipids are autocrine ligands for TLR2, which then triggers an inflammatory program in macrophages, via pathways dependent on MyD88. However, participation of lysophospholipids in this mechanism cannot be ruled out. In support of our hypothesis, when catalytic activity of MT-III is inhibited, increments in LD formation are abrogated [14]. In addition, the involvement of TLR2 in the release of the cytokines IL-1β and IL-10 induced by minimally modified LDL in macrophages has been previously described [46]. In conclusion, we provide evidence of a critical role for TLR2 and MyD88 adaptor molecule in mediating sPLA_2_-induced inflammatory responses in macrophages. We have shown for the first time that the TLR2 and Myd88 adaptor molecule are involved in cellular and molecular inflammatory processes triggered by the venom sPLA_2_ MT-III in macrophages, that include the production of eicosanoids and cytokines and the formation of LDs, as well as an increased expression of COX-2 and perilipin-2 mobilization and expression. Interestingly, these findings indicate that TLR2, via MyD88 adaptor molecule, links sPLA_2_s with LD and foam cell formation. Moreover, we speculate that saturated free fatty acids released by MT-III enzyme activity from phospholipids in the macrophage membrane, are autocrinally recognized by TLR2, thereby initiating an inflammatory signaling cascade in macrophages. Altogether, these findings amplify the current knowledge on the mechanisms involved in the stimulatory effects of Group IIA sPLA_2_s in macrophages, specially those that trigger inflammatory events, including foam cell formation, which is a hallmark of atherosclerosis. Such a mechanism may contribute to the identification of useful new targets for the development of novel therapeutic strategies for inflammatory diseases related to lipid imbalance where group IIA sPLA_2_s enzymes are involved.
