# Correction: Critical Role of TLR2 and MyD88 for Functional Response of Macrophages to a Group IIA-Secreted Phospholipase A_2_ from Snake Venom

**DOI:** 10.1371/journal.pone.0105241

**Published:** 2014-08-01

**Authors:** The PLOS ONE Staff

The affiliation for co-author, Dr. Jesús Balsinde, is incorrect. Please refer to the correct affiliation below:

The eicosanoid research division, Instituto de Biología y Genética Molecular, Consejo Superior de Investigaciones Científicas (CSIC), Universidad de Valladolid, 47003 Valladolid, Spain.
